# Continuation Rates of the 52-mg Levonorgestrel-releasing Intrauterine System according to the Primary Reason for its Use

**DOI:** 10.1055/s-0041-1726056

**Published:** 2021-05-12

**Authors:** Laura Miranda, Maria Helena de Sousa, Anibal Faundes, Cássia Juliato

**Affiliations:** 1 Universidade Estadual de Campinas, São Paulo, SP, Brazil; 2 Faculdade de Medicina de Jundiaí, Jundiaí, SP, Brazil

**Keywords:** levonorgestrel IUS, indication for use, continuation rate, amenorrhea, bleeding and spotting, counselling, sistema liberador de levonorgestrel (SIUS), indicação para uso, taxa de continuação, amenorreia, sangramento e microrragia intermenstrual, aconselhamento

## Abstract

**Objective** To evaluate whether continuation rates with the 52-mg levonorgestrel-releasing intrauterine system (LNG-IUS) up to 5 years after placement differed between women using the method exclusively for contraception and those using the device for medical reasons alone.

**Methods** A retrospective cohort study was conducted in a family planning clinic with 5,034 LNG-IUS users: 4,287 using the method exclusively for contraception and 747 for medical reasons alone. The continuation rate at 1 to 5 years of use was calculated by life table analysis.

**Results** Initially, the continuation rate was significantly higher in the contraception group: 85.8 versus 83.4 and 77.4 versus 76.0 per 100 women-years in the 1 ^st^ and 2 ^nd^ years of use, respectively. There were more discontinuations due to bleeding/spotting in the medical reasons group in the first two years. The discontinuation rate according to reason for use was not significantly different from the third to the fifth year of use. No women discontinued due to amenorrhea in either group.

**Conclusion** The continuation rate was significantly higher in the contraception group in the first two years of use. Amenorrhea was not a reason for discontinuation in either group, suggesting that counselling in this respect was adequate. Nevertheless, counselling could perhaps have been better with regards to the expected long period of bleeding and spotting in the first two years after placement.

## Resumo

**Objetivo** Avaliar a taxa de continuação até 5 anos de uso do sistema intrauterino liberador de 52-mg levonorgestrel por dia (SIU LNG) -IUS) é diferente entre mulheres que o usam exclusivamente como anticoncepcional que entre as que usam exclusivamente por razões médicas.

**Métodos** Estudo retrospectivo realizado em uma clínica de Planejamento Familiar 5.034 usuárias de SIU LNG, 4.287 que optaram pelo método apenas como anticoncepcional e 747 que o usavam somente por razoes médicas. A taxa de continuação de um até cinco ano foi calculada por meio de análise de tabela de vida

**Resultados** No início a taxa de continuação foi significativamente maior no grupo da anticoncepção: 85,8 versus 83,4 e 77,4 versus 76,0 por 100 anos-mulher no 1° e 2° ano de uso, respectivamente. Houve mais descontinuações por sangrado-manchado no grupo de razões médicas nos dos primeiros anos. A taxa de continuação não foi significativamente diferente desde o terceiro até o quinto ano de uso. Nenhuma mulher de ambos os grupos descontinuou por amenorreia.

**Conclusão** A taxa de continuação foi significativamente maior no grupo de anticoncepção durante os dos primeiros anos de uso. Amenorreia não foi motivo de descontinuação em ambos os grupos, sugerindo que a orientação a esse respeito foi adequada. Entretanto, a orientação referente ao longo período de sangramentos irregulares nos dois primeiros anos após a inserção, precisaria ser melhorado.

## Introduction

The 52-mg levonorgestrel-releasing intrauterine system (LNG-IUS) was developed by the Population Council's International Committee for Contraception Research (ICCR) in the 1980s as a contraceptive method. However, from the very beginning of its development, it was clear that the observed reduction in both the amount and duration of menstrual bleeding and the consequent improvement in hemoglobin levels constituted important non-contraceptive health benefits. 1 2 This finding led to subsequent approval of the method as a treatment for women with heavy menstrual bleeding (HMB). 3 The positive effect of this device in reducing pelvic pain in women with endometriosis, adenomyosis, and dysmenorrhea became evident a few years later and has been repeatedly confirmed. 4 5 Currently, the medical effects of the 52-mg LNG-IUS have become a major indication for its use, being as important as its role as a contraceptive.

In the initial multicenter evaluations of this device, the continuation rates were not very impressive: less than 60% at 2 years and 33% at 5 years of use. The main reason for discontinuation reported by users in the earlier trials was amenorrhea. 2 Conversely, this side effect would be most welcome for women using the method to treat HMB or dysmenorrhea.

Consequently, our hypothesis was that continuation rates with the LNG-IUS would be better in women using the device for medical reasons, particularly those with HMB, compared with women using it purely for contraceptive purposes. The objective of the present study was to evaluate whether there was any difference in continuation rates of the LNG-IUS up to 5 years after placement of the device between two different cohorts of women: a group using the method exclusively for contraception and another group using the device for medical reasons alone.

## Methods

This was a retrospective cohort study involving data collected systematically for the clinical records of all women initiating use of the 52-mg LNG-IUS (Mirena, Bayer Oy, Turku, Finland) at the family planning clinic of the Department of Obstetrics and Gynecology, School of Medical Sciences, Universidade de Campinas, Campinas, São Paulo, Brazil. The institution's internal review board approved the study protocol. No data were collected that would allow any of the women to be identified, thus ensuring the confidentiality of this information. Since this was a retrospective study based on data obtained from patients' medical records and because the identity of the women was maintained confidential, the requirement for informed consent was waived.

This clinic is one of the recipients of LNG-IUS devices provided at no cost by the International Contraceptive Access (ICA) Foundation. The clinic, then, offers the device to clients, also free of charge. Every woman who had decided to use the LNG-IUS at this clinic between January 3 ^rd^ , 2007 and June 30 ^th^ , 2015 was considered eligible for inclusion in the study.

Women who agreed to use the LNG-IUS for contraceptive reasons alone constituted the *contraception group* . These women were required to meet the following inclusion criteria: 1) age 14 to 50 years; 2) willing to use a LNG-IUS; 3) no desire to conceive within the next 12 months; 4) currently sexually active with a male partner; and 5) willing to return to the clinic at least once every 18 months to confirm that they continued to use the device and to register any possible event occurring during its use.

Women at the same clinic and during the same time period who opted to use the LNG-IUS for the sole purpose of treating or preventing a medical condition constituted the *medical reasons group* . These women were required to meet the following inclusion criteria: 1) age 14 to 50 years; 2) suffering from medical disorders such as HMB, having a hematological disorder, in use of anticoagulant drugs, or suffering from dysmenorrhea or endometriosis-associated pelvic pain; 3) willing to return to the clinic at least once every 18 months to confirm that they continued to use the device and to register the occurrence of any possible event during its use.

Despite requesting that they return after 18 months of use, the general policy of the clinic was followed, which is to inform women that they do not need to return to the clinic if they do not have any problem related to the method used, following recommendations of the medical literature. 6 7

The exclusion criteria were 1) women coming from another healthcare facility for the sole purpose of obtaining the device free of charge without being a client of this clinic, and 2) women requesting to use the LNG-IUS both for contraceptive purposes and for medical reasons. There were no active means of recording the follow-up of these women other than taking due note of every visit made by the participants to the clinic. Women who had come from other healthcare units or clinics for the sole purpose of obtaining the device and having it inserted were instructed to continue their follow-up at the clinic of origin, and, for that reason, were not included from this analysis.

The sample size was not calculated because no earlier studies were found on which to base estimations of expected differences between groups. The large number of women using the LNG-IUS at this clinic (over 4,000 in one group and over 700 in the other), however, allows us to be confident that the sample size was large enough to allow relatively small differences in the continuation rate to be identified, at least during the initial years of use.

## Statistical Analysis

Clinical records stored in Microsoft Excel were transferred to a database constructed using the SPSS statistical software package, version 20.0 (IMDB Corp., Armonk, NY, USA), which was used for the statistical analysis. Two independent data entry clerks input the data in duplicate, and any inconsistencies were reviewed and corrected.

The main outcome variable was the continuation rate at 1 to 5 years of use of the LNG-IUS, calculated by life table analysis. The predictor variable consisted of the reason for using the method: either exclusively for contraception or exclusively for medical reasons. The control variables or potential confounders were age at the time of LNG-IUS placement, number of living children, years of schooling, and marital status.

Continuation rates after 1, 2, 3, 4, and 5 years of use of the LNG-IUS exclusively for contraceptive purposes were compared with the respective rates in the group of women using the method for medical reasons alone. The sociodemographic characteristics of the women in each group were compared using Pearson chi-squared test or the chi-squared test with Yates correction.

The Cox multiple regression analysis was applied for each year of use, with five predictors/covariates to determine which were associated with the discontinuation rate. The backward method was used to select the variables. The significance level was established as 5% throughout the analysis.

## Results

A total of 5,034 women who had opted to use a LNG-IUS were included in this analysis, with 4,287 using the device exclusively for contraception and 747 exclusively for medical reasons. Many women were lost to follow-up, particularly in the 1 ^st^ year after insertion, with 1,542 losses. Subsequently, 770 women were lost to follow-up in the 2 ^nd^ year, 435 in the 3 ^rd^ year, 263 in the 4 ^th^ year, and 210 in the 5 ^th^ year of use. Loss to follow-up was the main reason for the decline in the number of subjects under observation over time, with the number of users falling to 2,889 in the 2 ^nd^ year, 1,882 in the 3 ^rd^ year, 1,304 in the 4 ^th^ year and 938 in the 5 ^th^ year of observation. The women in the contraception group were significantly younger, had had fewer children, and were more likely to have a stable partner than those in the medical reasons group. No statistically significant differences were found between the groups with respect to years of schooling ( Table 1 ).

**Table 1:** Sociodemographic characteristics of the sample according to the women's reason for using the levonorgestrel-releasing intrauterine system

Characteristics of users	Reason for use				
	Contraception only		Medical reasons only		*p* -value
	n	%	n	%	
Age (years) ( *n* = 5,045)					
≤ 24	629	14.6	37	4.9	< 0.001 a
25–34	2,124	49.4	222	29.7	
≥ 35	1,544	35.9	489	65.4	
Parity ( *n* = 5,037)					
1	2,190	51.0	302	40.6	< 0.001 a
2	1,633	38.0	283	38.1	
≥ 3	471	11.0	158	21.3	
Marital status ( *n* = 4,769)					
In a stable union	3,325	79.0	395	70.4	< 0.001 ^b^
No steady partner	883	21.0	166	29.6	
Schooling ( *n* = 4,750)					
≤ 12 years	3146	75.0	407	73.5	0.473 ^b^
≥ 13 years	1,050	25.0	147	26.5	

a Pearson chi-squared test; ^b^ Yates chi-squared test.

The continuation rates for the entire group of LNG-IUS users were 85, 77, 70, 64, and 58 per 100 women-years at 1, 2, 3, 4, and 5 years after insertion, respectively. During the first 2 years of observation, the continuation rate was significantly higher in the contraception group, compared with the medical reasons group ( Table 2 ). From the 3 ^rd^ to the 5 ^th^ years of use, this situation inverted, with a higher, although not significantly different, continuation rate in the medical reasons group ( Table 2 ).

**Table 2:** Cumulative continuation rate of the levonorgestrel-releasing intrauterine system over 1 to 5 years of use, according to reason for use ( *n* = 5,034)

Time (years)	Contraception only			Medical reasons only			*p* -value a
	Number of users end of the year	Continuation rate (%)	SE rate (%)	Number of users end of the year	Continuation rate (%)	SE rate (%)	
1	4,287	85.8	0.6	747	83.4	1.5	0.002
2	2,451	77.4	0.8	438	76.0	1.8	0.045
3	1,559	70.1	0.9	323	71.3	2.0	0.121
4	1,059	63.2	1.1	245	67.7	2.2	0.166
5	752	57.5	1.2	186	61.2	2.5	0.186

Abbreviations: N, Number of women at the start of the period analyzed; SE, standard error.

a Wilcoxon-Gehan test.

Following the Cox multiple regression analysis, the significant association between using the device for contraceptive purposes alone and a higher continuation rate persisted for the first 2 years of use, while older age was associated with a significantly higher continuation rate at each of the 5 years of observation ( Table 3 ).

**Table 3:** Characteristics significantly associated with continuation rate after 1 to 5 years of use of the levonorgestrel-releasing intrauterine system following the Cox multiple regression analysis ( *n* = 4,728) a

Time of use/ Characteristics	Coef.	SD coef.	*p* -value
Up to one year			
Age (years)	0.020	0.004	< 0.001
Reason for use (contraception)	0.281	0.074	< 0.001
Up to 2 years			
Age (years)	0.022	0.004	< 0.001
Reason for use (contraception)	0.177	0.074	0.017
Up to 3 years			
Age (years)	0.021	0.004	< 0.001
Up to 4 years			
Age (years)	0.022	0.004	< 0.001
Up to 5 years			
Age (years)	0.023	0.004	< 0.001

a Data missing in 317 cases. Covariates included in the analysis: age (years); parity (≤ 1:0 /> 1:1); marital status (in a stable union: 0 / no steady partner: 1); schooling (up to high school: 0 / at least some college education: 1); reason for use (contraception alone: 1 / medical reasons alone: 0).

The main reason for discontinuation was expulsion of the device, followed by bleeding/spotting, and desire to conceive. Discontinuations due to acne or for other steroid-related reasons were few. No women discontinued use of the device because of amenorrhea in either of the two groups ( Table 4 ).

**Table 4:** Cumulative discontinuation rates up to 5 years of use of the levonorgestrel-releasing intrauterine system according to reasons for discontinuation and indication for using the method

Cause of discontinuation	Indication for using the LNG-IUS				
	Contraception		Medical reasons		*p* -value a
	N	(%)	N	(%)	
After 1 year	4,287		747		
Expulsion		7.9		11.6	< 0.001
Bleeding/spotting		0.8		1.3	0.004
Pain		1.4		0.8	0.320
Amenorrhea		0.0		0.0	
Acne		0.2		0.2	0.204
Desire to conceive		1.0		0.4	0.004
Other		3.5		2.9	0.061
After 2 years	2,451		438		
Expulsion		10.5		15.2	< 0.001
Bleeding/spotting		1.4		2.1	0.023
Pain		2.4		1.8	0.264
Amenorrhea		0.0		0.0	
Acne		0.7		0.2	0.194
Desire to conceive		4.0		1.7	0.001
Other		5.8		5.2	0.610
After 3 years	1,559		323		
Expulsion		11.9		16.3	< 0.001
Bleeding/spotting		1.8		3.2	0.057
Pain		3.2		1.8	0.257
Amenorrhea		0.0		0.0	
Acne		1.0		0.6	0.199
Desire to conceive		8.5		4.2	< 0.001
Other		7.7		6.5	0.807
After 4 years	1,059		245		
Expulsion		13.3		17.9	< 0.001
Bleeding/spotting		2.1		3.2	0.087
Pain		4.3		2.3	0.253
Amenorrhea		0.0		0.0	
Acne		1.7		0.6	0.227
Desire to conceive		11.7		5.5	0.001
Other		10.6		7.5	0.557
After 5 years	752		186		
Expulsion		13.9		18.9	< 0.001
Bleeding/spotting		2.6		5.1	0.113
Pain		5.0		2.3	0.238
Amenorrhea		0.0		0.0	
Acne		2.0		0.6	0.236
Desire to conceive		14.5		6.6	< 0.001
Other		14.4		12.0	0.489

a Chi-squared test with Yates correction.

The discontinuation rate due to bleeding/spotting was greater in the medical reasons group compared with the contraception group throughout the entire 5 years of use; however, the difference was only statistically significant in the first 2 years of observation. There were significantly more expulsions throughout the 5 years of observation in the group of women using the method solely for medical reasons. Conversely, women using the method for contraception alone were significantly more likely to discontinue because they wanted to become pregnant compared with those using the device for medical reasons, throughout the entire 5-year study period ( Table 4 ).

## Discussion

The present study shows that in this population of women living in a large Brazilian city of more than a million inhabitants and opting to use the LNG-IUS, amenorrhea was no longer a reason for discontinuation. This is largely due to very good counselling, particularly with respect to informing potential candidates that the possibility of experiencing reduced bleeding, fewer bleeding episodes, or no bleeding at all is high. Consequently, women who were not willing to stop menstruating on a regular basis probably chose not to use the method, while those who elected to use the LNG-IUS were no longer concerned about becoming amenorrheic. Similar results were found in a previous study conducted in the same city. 8 On the other hand, the inconvenience of unpredictable bleeding or spotting became an important reason for discontinuation, particularly in the case of women using the device for medical reasons usually related to HMB or dysmenorrhea.

The present continuation rates were better than those reported in the initial evaluations of the device, when less than 60% of users continued with the LNG-IUS after 2 years, compared with 77% in the present study. 2 Rates were also better than those reported from a more recent multicenter study conducted by the World Health Organization, particularly after 3 years (61.8 versus 70 per 100 women-years) and after 5 years of use (47.5 versus 58 per 100 women-years). 9 The discontinuation rate in this study population was, however, similar to the rate found in the US-based Contraceptive Health Research Of Informed Choice Experience **(** CHOICE) study, which reported continuation rates of 88% (versus 85%) at 1 year; 79% (versus 77%) at 2 years; 62.5% (versus 64%) at 4 years, and 51.7 (versus 58%) at 5 years. 10 11 12 In fact, the contraceptive services offered in our clinic parallel those provided in the US-CHOICE study in that all the methods, including the LNG-IUS, were supplied to the women at no cost.

One possible explanation for the relatively good results found in the present study is that this clinic is one of the most experienced in the world in the use of this method, having worked with the LNG-IUS since 1978. 13 Hence, the healthcare professionals working here have a vast experience in counselling women interested in using the device. It would appear, however, that the explanations provided regarding the long period preceding amenorrhea, when the woman could experience bothersome spotting or even bleeding, are insufficient. The LNG-IUD user should be aware of this and be patient, knowing that these bleeding irregularities will pass with time. This is crucial for women using the method for medical reasons, whose expectation of avoiding menstruation is greatest.

The significantly higher expulsion rate in the group of women using the device as a treatment for HMB was expected and is in agreement with previous findings in this same clinic showing that expulsion is three times more likely in these women compared with those using the device for contraception alone. 14 Most probably, some of these women had the device inserted during an episode of HMB, when the uterine muscles are irritated and may cause uterine contractions that could result in expulsion.

The higher discontinuation rate due to a desire to conceive in the group of women using the device for contraceptive purposes alone was expected, since the women using the method for medical reasons alone were older and had already had more children compared with the contraception group.

The main limitation of the present study is the relatively high rate of loss to follow-up, which reached 26%. Another study conducted with this same population showed that loss to follow-up was associated with a large proportion (> 25%) of college-educated LNG-IUS acceptors, a situation that is not typical of the clientele in this public healthcare clinic. 15 Better-educated women with higher income levels are less dependent on the care provided at this family planning clinic and are better prepared to act according to the information given at insertion; thus, follow-up visits would not have improved performance if there were no complications during the use of the LNG-IUS. This, however, results in a high rate of loss to follow-up, which would potentially bias the results. In case the main reason for not returning to follow-up was the understanding that these visits were not required for the successful use of the method, it is possible that the continuation rate could have been better. Although such hypothesis is impossible to test, the opposite possibility, that users had the device removed in another clinic, is even less plausible.

The main lesson learned here is the need to provide careful and accurate information prior to the insertion of the device, focusing on the possibility of bothersome spotting and bleeding, which, although insignificant in terms of quantity, is unpredictable insofar as both timing and duration are concerned. This is particularly relevant in the case of women using the method for medical reasons if the expectation is to be free from menstruation with the use of the device. Although menstruation will not be painful, frequent bleeding, even in small quantities, is unacceptable to many women and they have the right to be adequately informed of this problem before deciding whether to use the device.

## Conclusion

Although these results originate from one clinic within the Brazilian public healthcare service network, we believe that the recommendation may equally apply to any other sites where the LNG-IUS is available. Providing adequate counselling to women selecting a contraceptive device is an important component of the quality of care, and, in this case, information on the expected bleeding characteristics in the 1 ^st^ year of use of the LNG-IUS should be a focus of counseling everywhere. We expect that by improving this aspect of counseling in our clinic, the current relatively good continuation rate can further improve in the future.
